# Timing matters: sonar call groups facilitate target localization in bats

**DOI:** 10.3389/fphys.2014.00168

**Published:** 2014-05-12

**Authors:** Ninad B. Kothari, Melville J. Wohlgemuth, Katrine Hulgard, Annemarie Surlykke, Cynthia F. Moss

**Affiliations:** ^1^Auditory Neuroethology Lab, Psychology, University of MarylandCollege Park, MD, USA; ^2^Biology, University of Southern DenmarkOdense, Denmark

**Keywords:** echolocation behavior, sonar call timing, active sensing, spatial perception, target tracking

## Abstract

To successfully negotiate a cluttered environment, an echolocating bat must control the timing of motor behaviors in response to dynamic sensory information. Here we detail the big brown bat's adaptive temporal control over sonar call production for tracking prey, moving predictably or unpredictably, under different experimental conditions. We studied the adaptive control of vocal-motor behaviors in free-flying big brown bats, *Eptesicus fuscus*, as they captured tethered and free-flying insects, in open and cluttered environments. We also studied adaptive sonar behavior in bats trained to track moving targets from a resting position. In each of these experiments, bats adjusted the features of their calls to separate target and clutter. Under many task conditions, flying bats produced prominent *sonar sound groups* identified as clusters of echolocation pulses with relatively stable intervals, surrounded by longer pulse intervals. In experiments where bats tracked approaching targets from a resting position, bats also produced sonar sound groups, and the prevalence of these sonar sound groups increased when motion of the target was unpredictable. We hypothesize that sonar sound groups produced during flight, and the sonar call doublets produced by a bat tracking a target from a resting position, help the animal resolve dynamic target location and represent the echo scene in greater detail. Collectively, our data reveal adaptive temporal control over sonar call production that allows the bat to negotiate a complex and dynamic environment.

## Introduction

How do animals process, organize and retrieve information from a rich and complex environment? Furthermore, how is this information integrated with motor programs to support perceptually-guided behaviors? The active sensing system of the echolocating bat presents an opportunity to address these questions. The bat produces ultrasonic signals and uses information carried by echoes to detect, localize and discriminate objects in the environment. It is well established that echolocating bats adapt the duration, spectrum, directional aim and timing of sonar signals in response to information extracted from echoes (Griffin, 1958; Jen and McCarty, 1978; Petrites et al., 2009; Moss and Surlykke, 2010). Past research has considered the functional importance of adaptive control of bat sonar call parameters (pulse duration, interval, spectrum, and beam aim) in the context of behavioral tasks, such as prey capture and obstacle avoidance, and the environment in which the bat operates, e.g., open space, forest edge, or within dense vegetation (Griffin et al., 1960; Kalko and Schnitzler, 1989, 1993; Simmons et al., 1979; Surlykke and Moss, 2000; Siemers and Schnitzler, 2004; Moss et al., 2006; Jones and Holderied, 2007). Layered on the adaptive changes in sonar signal parameters is the temporal patterning of calls, but the functional importance of this behavior is not well understood. Here, we compare the global temporal patterning of sonar vocalizations in different situations from both field and laboratory studies of the big brown bat, *Eptesicus fuscus*, with the goal of advancing our understanding of the environmental and task conditions that influence the bat's control over the timing and grouping of calls.

When the big brown bat is hunting and searching for prey in an open habitat, long, shallow FM (frequency modulated) signals facilitate target detection by concentrating sound energy in a narrow frequency band over an extended period of time. During target approach and interception, the bat emits broadband vocalizations that support target localization in azimuth, elevation and range, as each frequency band in the echo provides a time marker for its arrival at the bat's ears (Moss and Schnitzler, 1995; Surlykke and Moss, 2000). In addition, the FM bat actively adjusts the duration of signals to avoid overlap of sonar emissions and echoes, and modifies sonar call intervals to receive echoes from one sonar emission before producing the next (Kalko, 1995; Wilson and Moss, 2004; Surlykke et al., 2009b).

The bat's adjustments of sonar signal repetition rate and duration are tied to target range; however, echolocation call parameters also depend on the bat's azimuth and elevation relative to a selected prey item, and most importantly, its plan of attack. If a bat approaches an insect, flies past it and returns to intercept it, the temporal patterning of the bat's signals are distinctly different from those produced by the bat if it flies directly to attack the prey (Moss and Surlykke, 2001; Moss et al., 2006). Thus, the temporal patterning of the bat's echolocation signals provide explicit data on its adaptive motor commands to actively probe objects in the auditory scene.

In more challenging behavioral contexts, the bat produces clustered groups of vocalizations, previously termed sonar “strobe groups,” because three or more signals within such a group typically have relatively stable pulse intervals (5% tolerance), and are flanked by calls with larger intervals (Moss et al., 2006). Here we refer to these call groups as sonar sound groups, to include the production of two, as well as three or more calls emitted in clusters, surrounded by longer pulse intervals (1.2 times the mean interval within the call cluster). For call pairs, or doublets, it is not relevant to consider the stability of call intervals, and hence the term “strobe” would not apply. Petrites et al. (2009) and Hiryu et al. (2010) have defined “strobe groups” slightly differently. However, the basic concept of a group of sounds with near constant pulse intervals, surrounded by calls with larger intervals remains the same.

A previous study of the vocal behavior of echolocating bats flying in environments with acoustic clutter reported that big brown bats produce pairs of vocalizations, or sound doublets, flanked by calls with longer intervals (Hiryu et al., 2010). Furthermore, these pairs of vocalizations showed specific and reliable differences between the frequency content of individual calls. The big brown bat altered the frequency of the second vocalization in the doublet with respect to the first, and it was hypothesized that such spectral adjustments permit the disambiguation of echo cascades from the first and second vocalization in the pair. The change in frequency across vocalizations in a sonar sound doublet suggests that the bat combines echo information from the first and second calls to represent a complex environment. In this way, the bat may be integrating echo information over a sequence of acoustic snapshots (see Moss and Surlykke, 2001).

Other studies of bats foraging in the laboratory have highlighted the temporal patterning of sonar calls produced by bats. Moss and Surlykke (2001) and Moss et al. (2006) reported that the prevalence of sonar sound groups was greater when the big brown bat foraged close to background clutter than in the open room. They observed that bats tended to produce sonar sound groups when selecting a target, changing the direction of the flight path, or when the bats were in close proximity to obstacles. These observations led to the hypothesis that sonar sound groups have immediate consequences for the bat's perception of space and are used in planning a flight trajectory that requires a more detailed and updated estimate of target localization (Moss and Surlykke, 2001; Moss et al., 2006). These ideas demand a more complete investigation, and in this article, we further consider the echolocating bat's temporal control of sonar calls to represent the environment in a variety of habitats and behavioral contexts.

Here we compare echolocation behaviors in several distinct studies of the big brown bat (*E. fuscus*) from both the field and the laboratory, and under different environmental and task conditions. We re-examine data from our previously published studies (Surlykke and Moss, 2000; Moss and Surlykke, 2001; Ghose and Moss, 2006; Moss et al., 2006; Ghose et al., 2009; Surlykke et al., 2009b), along with newly collected data. Our focus is on the bat's temporal control over sonar call production, and we consider a variety of factors that may contribute to the timing of bat sonar calls, including wing beat, background clutter, target motion, and bat flight trajectory. We hypothesize that for more demanding spatio-temporal localization tasks, the echolocating bat actively adjusts the timing of calls to increase the reliability and/or resolution of spatial and temporal information acquired from echoes.

## Methods

**Figure 1:**
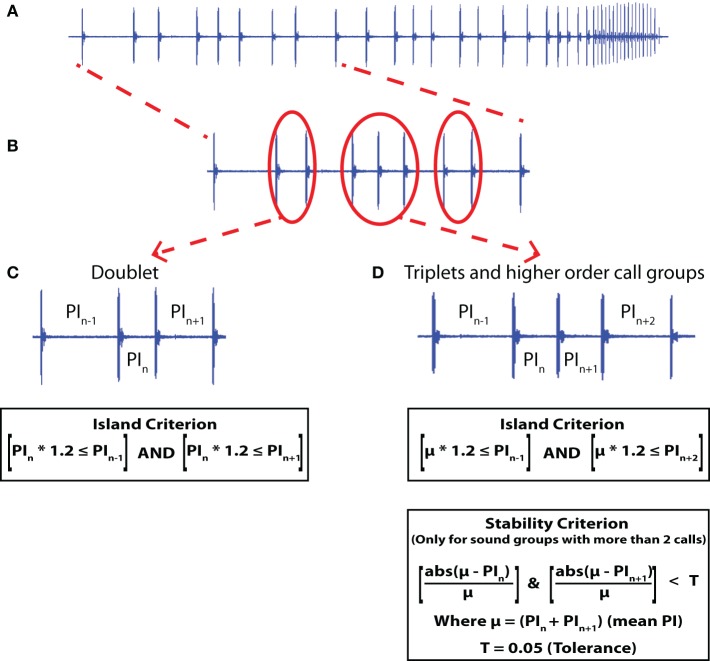
**Sonar sound groups. (A)** A sonar call sound stream from a bat tracking a tethered meal worm following the Simple Motion (SM) trajectory. **(B)** Doublets and Triplet sound groups. **(C)** A doublet is identified by the PI of the calls at either end of the doublet being at least 1.2 times larger than the PI of the doublet (Island Criterion). **(D)** Higher order sonar sound groups are identified by a stable PI within the call group (Stability Criterion). The stable PI is indicated here as the mean (μ) and the PI is considered stable if all the PIs within the group are within a tolerance of ±5% (T) of the mean PI. Also, the PI of the calls at either end should be at least 1.2 times the mean PI of the calls in the sound group (Island Criterion). Here the example given is of a triplet sound group.

Audio recordings were taken from echolocating big brown bats, behaving in the lab and the field, and the focus here is on the timing of sonar call production. Microphone and data acquisition systems were specific to the field and lab studies and are detailed below. Previously, Moss and Surlykke (2001) and Moss et al. (2006) defined sonar sound groups as clusters of three or more vocalizations which occur with a near constant PI (within 5% error with respect to the mean PI of the sound group), and are flanked by calls with a larger PI at both ends (at least 1.2 times larger). We refer to the property of sound groups flanked by calls with larger PI at both ends as meeting an Island Criterion (see Figures 1C,D). The terminology Island Criterion refers to the temporal isolation of sonar sound groups within the ongoing stream of sonar vocalizations. Additionally, we term the near constant PI within a sound group as meeting a Stability Criterion (see Figure 1D). Since the Stability Criterion cannot be defined for sonar call doublets which are pairs of sonar sounds produced with a short PI compared with surrounding calls, sonar sound doublets are characterized solely by the Island Criterion (see Figure 1C). The Island Criterion was used in the current study, to characterize a broader scope of temporal call patterning, and we collectively refer to clustered signals as Sonar Sound Groups. Hence, sonar sound groups with three or more clustered sonar sounds satisfy both the Island Criterion and the Stability Criterion, whereas the sonar sound doublets only satisfy the Island Criterion.

### Field recordings

**Figure 2:**
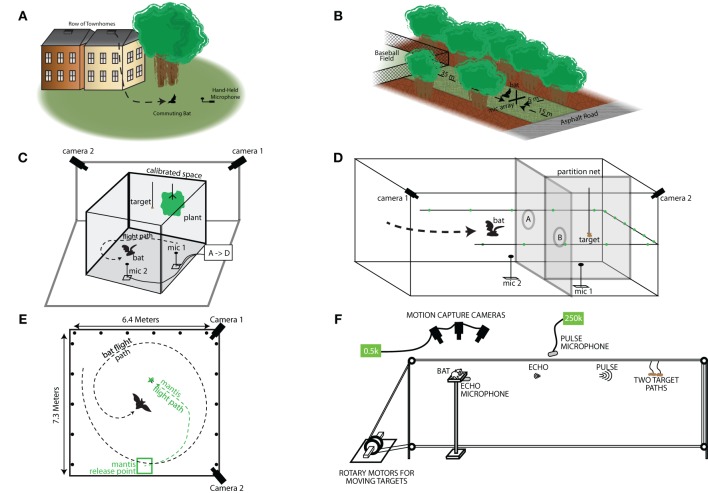
**Field and laboratory experimental setups. (A)** Schematic of the Rockville, MD field site. Bats were exiting from behind a slatted vent near the roof on the side of a town home. This town home was at the end of a row of town homes that opened up onto a small field with a few trees. The bats' vocalizations were recorded as they flew out of the house and onto the field. **(B)** Layout of the Lake Artemesia, MD field site. The recording site was a narrow corridor of grass between trees, bounded at one end by a baseball field and at the other by a paved road. The bats were recorded with a microphone array placed at the road-end of the corridor. **(C)** Laboratory setup for catching tethered mealworms in the presence of clutter. The clutter was a fern-like artificial plant hung from the ceiling, and mealworms were tethered to the ceiling at varying distances from the plant. Two cameras in the corners of the room capture 3D flight trajectory data, while microphones on the floor recorded sonar vocalizations. **(D)** Experimental setup for the net-hole experiment. The room was portioned as shown into three sections. The mealworm was hung in one of the two smaller sections on the right end of the room, and the bat flew through either hole “A” or hole “B” do catch the mealworm. Behavioral measurements as described above (i.e., flight path, vocalizations, beam shape) were collected. **(E)** Top-down view of the laboratory setup for the flying mantis experiment. The mantis was released from a platform, and the bat was released by the researcher elsewhere in the room. Two cameras recorded the 3D flight path, while microphones on the floor and walls (round marks) recorded the sonar vocalizations, and beam shape, respectively. **(F)** Schematic of the setup for the platform tracking experiment. A bat is trained to sit on a platform and track a tethered mealworm that is moved in the range axis with a computer controlled set of rotary stepper motors. The bats' vocalizations and returning echoes are recorded by ultra-sonic microphones in front and underneath the bat, respectively. Motion capture cameras collect ear and head movement data.

Field recordings of *E. fuscus* were taken at two different sites (Figure 2; sites A and B). Recordings at site A were carried out in the months of August and September of 1999, when bats were commuting from a roost in Rockville, MD, U.S.A. The bats emerged from their roost which was a small opening in the roof of a town house. The opening faced a group of trees, and a hand held ultrasound microphone was used to record the vocalizations as the bats flew out (Figure 2A). Further details of the methods and the site of the 1999 field recordings are reported in Surlykke and Moss (2000). Recordings were made at Site B in the month of May, 2013. Site B was located at Lake Artemesia, MD and can be briefly described as a rectangular open space (approximately 15 × 30 m) flanked by a baseball field and a deserted road on either end of its longer dimension and a thicket of trees and a small creek on opposites sides of its narrower dimension (Figure 2B). The setup at Site B consisted of 9 G.R.A.S. ¼ microphones placed in a cross-shaped array, 6 on a horizontal line and 2 above and 1 below the center microphone forming a 4 microphone vertical line. The horizontal microphones were placed from left to right at 0, 1.36, 2.70, 3.60, 4.50, and 6.11 m and the vertical microphones (with the 4th microphone at 3.60 m as center) were placed 2.85 and 1.15 m above and 0.57 m below the horizontal line. The amplified (Avisoft power modules) sounds were digitized, Avisoft USGH 1216 at 300 kHz sampling rate and stored on a laptop computer. We recorded 4 s files, 2 s pre-trigger and 2 s post-trigger. Triggering occurred when a feeding buzz was heard on a D240x Peterson bat detector. The microphones were calibrated before and after each recording session with a GRAS 42 AB sound calibrator.

### Recordings from free flying *E. fuscus* in the laboratory

Here we describe three different experiments, in which flying bats captured stationary (tethered mealworm) and moving insect (free-flying praying mantises) targets in a closed laboratory flight room, and in some studies in the presence of obstacles. The data presented here have been analyzed to examine and compare the bat's production of sonar sound groups under a variety of conditions. In all of these laboratory studies, bats flew freely in a large flight room, with walls and ceiling lined with acoustic foam (Sonex 1), and a carpeted floor. Two high speed Kodak MotionCorders (240 frames/s) or Photron video cameras (250 frames/s) recorded the bat's flight behavior under IR illumination, and the stereo video data were used to reconstruct the bat's 3D flight path within a calibrated volume in the room (Figures 2C–E). The bat's echolocation signals were recorded with two Ultrasound Advice microphones positioned on the floor and digitized with an IoTech 512 Wavebook at a sample rate of 240 kHz/channel. Only the data 3 s prior to the time when the bat captured or hit the tethered mealworm were analyzed and presented here.

#### Bats taking tethered insects in the laboratory under different clutter conditions

Bats were trained to take mealworms from a tether in an open (uncluttered) flight room. Clutter was introduced by an artificial houseplant, resembling a fern, approximately 80 cm in diameter and 50 cm high, hanging from the ceiling at the same elevation as the tethered mealworm. Trials were run with the tethered insects presented in an open room and at different distances from the vegetation, ranging from 10 to 40 cm. The setup is shown in Figure 2C. For more details, refer to Moss et al. (2006).

#### Obstacle avoidance task and prey capture in the laboratory

A mist net was used to divide the flight room into two partitions. One side of the room was further subdivided with a mist net to create two sub-compartments. A tethered mealworm was hung randomly in either of the two sub-compartments, and bats were trained to search for the tethered mealworm, and then fly through an opening in the mist net to collect its food reward in the sub-compartment where it was presented (as shown in Figure 2D). This task forced the bat to find the food reward behind the mist net and negotiate the obstacle (opening in the net) to collect the reward, hence requiring goal-oriented behavior in a complex environment (For further details, see Surlykke et al., 2009b).

#### Pursuit and capture of free-flying insects in the laboratory

Bats were trained to capture a freely flying praying mantis. Figure 2E shows the experimental setup with an example bat and mantis trajectory. The bat was released from different locations in each trial while the mantis was released from the same location. The hearing of the praying mantis was impaired by applying Vaseline to its midline ear (Triblehorn et al., 2008), and therefore the insect continued to fly when the bat produced ultrasonic signals which would otherwise trigger a dive response by the mantis. This experiment enabled us to study the sonar call production behavior of bats in an insect-tracking task. For more details, refer to Ghose and Moss (2006) and Ghose et al. (2009).

### Lab recordings of *E. fuscus* tracking a target while resting on a platform

**Figure 3:**
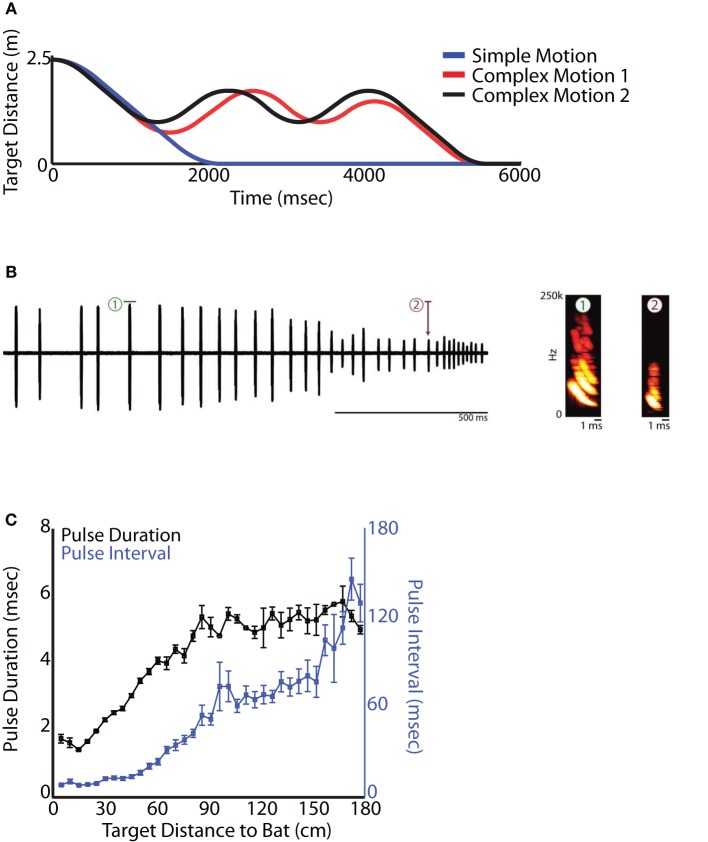
**Platform experiment. (A)** Distance vs. time for each type of target motion of the tethered mealworm. The blue line represents simple motion in only one direction, while the red and black lines are the more complicated, back-and-forth motions. **(B)** Left, example oscillogram of a sequence of vocalizations produced by a bat tracking a tethered mealworm in the setup shown in **(A)** moving in the simple motion trajectory. Right, spectrograms of the pulses highlighted by the red and green boxes on the left demonstrating the stereotyped changes in duration and frequency that are correlated with target distance. **(C)** Quantification of changes in pulse duration and pulse interval as a bat tracks a moving target on the setup shown in **(A)**.

Big brown bats (*E. fuscus*) were trained to sit on a platform and track a moving food reward (mealworm—Figure 2F). The food reward was tethered and suspended from a rectangular loop of fishing line with pulleys on 3 corners, and a rotary servo motor (Aerotech BMS60 brushless, slot-less rotary servo motor attached to an Ensemble MP10 motor controller) on the fourth corner that drove the fishing line in either direction (see Figure 2F). The rotary stepper motor was programmed via a computer interface through Matlab (2012a), controlling the velocity, acceleration, deceleration, and the distance the food reward traveled. This method engaged the bat in naturalistic sonar tracking behavior, while also allowing the experimenter precise control over the target motion with respect to the bat, which is not possible in free flight studies. This setup moved the target along the range axis on a straight line toward the bat. Because the bats were resting on the platform, the timing of calls would be coordinated with respiration but not influenced by wing beat (Wong and Waters, 2001; Wilson and Moss, 2004; Koblitz et al., 2010). Bat sonar vocalizations were collected using two Ultrasound Advice UM3 microphones (M1 and M2 in Figure 2F) and were digitized using a National Instruments A/D PCI card interfaced with Matlab (2012a). Two high speed infrared Phantom Miro cameras and 3 infrared Vicon Motion tracking cameras were used to track the head and pinnae movements of the bats. The Aerotech Servo motors, audio capture, high speed video and Vicon motion tracking cameras were all synchronized using the a single TTL trigger pulse generated via the Matlab-National Instruments A/D interface. Data analysis from the high speed video and Vicon motion tracking systems is not presented here. Initial stages of this task involved clicker training to condition the bat to associate a sound with the delivery of a food reward; the experimenter then slowly moved the food reward by hand while the bat used echolocation to track its position. Once the bat learned to track the food reward using echolocation, the insect was hung from the fishing line and initially moved small distances with the rotary stepper motor system. As the bat learned the task, the total target distance was increased to 2.5 m. During training, a single type of target motion was used: The target started at a distance of 2.5 m, accelerated at a rate of 7 m/s^2^, traveled a distance of approximately 2 m with constant velocity of 4 m/s (mimicking the approximate flight velocity of a bat during the approach phase (Hayward and Davis, 1964) and then decelerated at a rate of 5 m/s^2^. We refer to this motion as Simple Motion (SM). The end of the trial was marked when the tethered mealworm reached the bat. The bat would generally take the mealworm in the mouth and in the event it missed, the bat was then rewarded by hand. Additionally, catch trials were introduced, where the mealworm was stopped before it reached the bat to make sure that the bats were not just echolocating at random. Most trained bats would stop echolocating as soon as the mealworm stopped. The movement of the target with respect to the stationary bat is shown in Figure 3A. Figure 3B shows an example sonar recording of a bat tracking a mealworm. Sonar call spectrograms of an approach call (marked red) and a feeding buzz call (marked by green) are also shown. As previously demonstrated by Aytekin et al. (2010), well-trained bats actively adapt sonar PI according to the distance of the target (see Figure 3C). Once the bat became skilled at the SM tracking task, two novel types of target motion were introduced to the bat. We refer to these target motions as Complex Motions 1 and 2 (CM1 and CM2, respectively). In the novel complex motion trajectories, the target first moved toward the bat, after which it oscillated back and forth before finally reaching the bat. The target displacement relative to the stationary bat is shown in Figure 3A (Complex 1–red, Complex 2–black). The different parameters of the Simple Motion and two Complex Motions are shown in Table 1.

**Table 1:** **Motion parameters for each type of target motion a bat was presented with for the platform tracking experiment**.

**Trial type**	**Forward velocity**	**Backward velocity**	**Acceleration**	**Deceleration**	**Total motion time**
Simple motion	4 m/s	NA	7 m/s^2^	5 m/s^2^	1.8 s (approx.)
Complex motion 1	4 m/s	3.5 m/s	10 m/s^2^	10 m/s^2^	5.3 s (approx.)
Complex motion 2	4 m/s	3.5 m/s	10 m/s^2^	10 m/s^2^	5.5 s (approx.)

The main focus of this experiment was to test the hypothesis that the big brown bat actively produces clustered sonar sound groups to resolve spatial location when target trajectory is uncertain. In order to introduce target motion uncertainty, trial types (CM1, CM2, and SM) were randomized. Within the random presentation of trajectory types, a sequence of CM (1 or 2) followed by two or three SM trials, was presented. All analysis was performed on entire trials of the sequence of CM and SM trials.

## Analysis methods

Recorded sonar vocalizations were analyzed using custom written Matlab routines. Examples of a doublet and triplet sound groups are shown in Figure 1 and the criteria to identify sonar sound groups is illustrated. Individual details of the analysis for each experiment are given below.

### Flight trajectory analysis

In the field at site B, the 3D position of free-flying bats was computed based on arrival time differences at the nine microphones in the array using cross-correlation and then computing the position based on the sound emission times and triangulating (Surlykke et al., 2009a). The 3D position of the bat in the laboratory was calculated by using a calibrated region of overlap from the two high speed video recordings (Moss et al., 2006).

### Analysis of sonar signals produced by bats

The emitted sounds were analyzed using custom Matlab software to relate sound features, i.e., pulse timing, duration, and interval, to the bat's 3D position and distance to targets and obstacles. For more details of the sonar vocalization analysis in bat flight experiments, please refer to Ghose and Moss (2006); Moss et al. (2006); Ghose et al. (2009), and Surlykke et al. (2009b).

## Results

### Temporal control of echolocation signals produced by bats in the field

**Figure 4:**
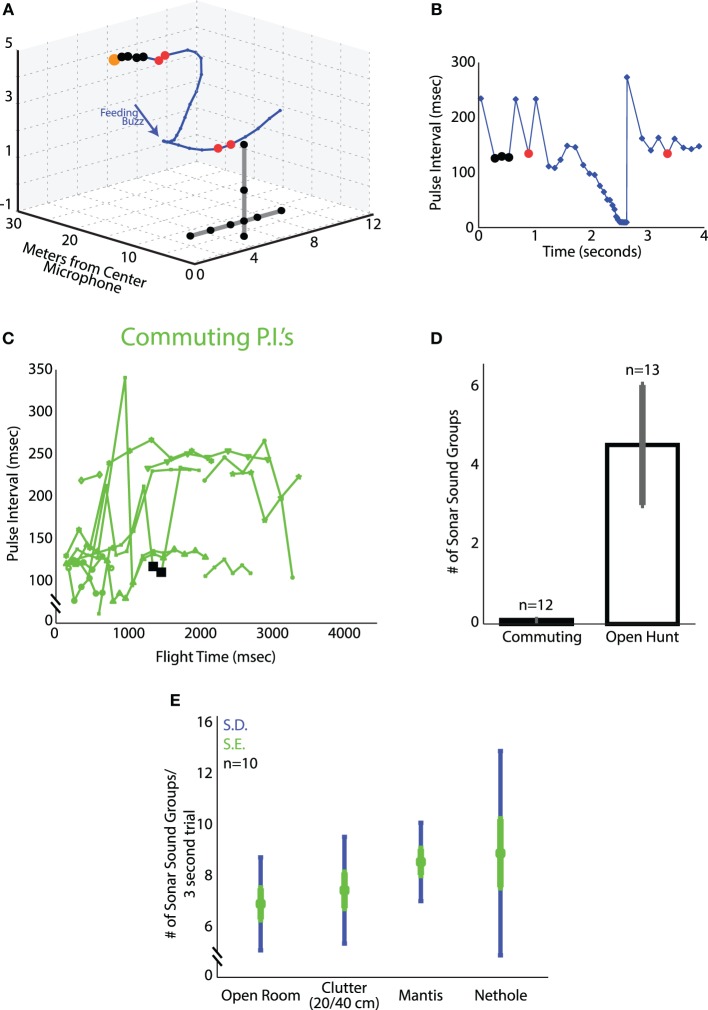
**Sonar sound groups under varying conditions. (A)** One trial plotted in 3D from the Lake Artemesia field site. The bat's flight path is shown in blue, and timing of the vocalizations with blue dots. Black dots highlight vocalizations in a 4 call sound group, calls marked in red are 2-call sound groups (sonar call doublets). The microphone array is shown in black. **(B)** Time vs. pulse interval for the trial shown in **(A)**. As in **(A)**, P.I.'s marked with black are 4 call sound groups, and those with red dots are sonar call doublets. **(C)** Time vs. pulse interval for the recordings of commuting bats at Rockville, MD. Only one sequence of vocalizations (shown in black squares) qualified as a sound group by our definition. The low (around 120 ms) and high (around 240 ms) PIs correspond to emitting a call per wing beat or only for every second wingbeat respectively. Sometimes the bats skipped two wingbeats and PI became even longer, around 350 ms. **(D)** Number of sound groups uttered per trial for the commuting bats at Rockville, MD; and the hunting bats of Lake Artemesia, MD. **(E)** Average number of sound groups per trial in the four laboratory flight experiments (clutter, nethole, mantis, open room). Green errorbars denote the standard error, blue the standard deviation.

Comparing bat echolocation patterns in the field and lab allows one to evaluate natural and artificial constraints on behavior. Here we report on the natural sonar behavior of big brown bats in the field as they (i) commuted from a roost (Site A) or (ii) foraged (Site B). Vocalizations recorded in the late evening when bats emerged from their roost were classified as “commuting sonar calls.” After bats flew out of their roost, they flew mainly in one direction and showed no circling around the roost area. No feeding buzzes were recorded in this setting, indicating that bats were not foraging immediately after flying out of their roost. Big brown bats are generally known to fly to foraging sites away from their roosts, where they find a high density of prey. The roosting sites are often found in locations, which are safe for the bats and their young, such as man-made structures, caves, mines as well as tree cavities (Brigham and Fenton, 1986; Agosta, 2002). Vocalizations recorded at foraging sites were classified as “foraging sonar calls.” The bat's flight and acoustic behavior during foraging was distinct from that observed in commuting animals. Foraging bats typically circled in a restricted area, following a relatively stereotyped trajectory, in contrast to the commuting trajectories which were straight in one direction. Many recordings at site B contained terminal buzzes, indicating that the bats were actively hunting. Figure 4A shows a typical trajectory of a bat while it was foraging at site B. Figure 4B shows the corresponding sonar pulse interval (PI) plot. Each marked point (in blue) on the PI plot and the 3D trajectory in Figures 4A,B shows a sonar vocalization. Sonar sound groups are marked in red (doublets) and black (sound groups with several sonar calls) solid circles on each plot. The first 3 black solid circles in Figure 4B (and corresponding 4 black solid circles Figure 4A) indicate a sound group, which consists of four calls in a series. Similarly the first and second red solid circles are doublets (and the corresponding red solid circles in Figure 4A are the doublet vocalizations). The sonar sound groups with two calls (red) and four calls (black) have been marked in different colors for illustration purposes. Figure 4C shows the PI plots of sound recordings at Site A when the bats were flying out of their roosts and commuting. Sonar sound groups were rarely observed in commuting bats (see one exception marked by black squares) and no feeding buzzes were recorded at site A. Figure 4D compares the mean number of sonar sound groups recorded when the bats were commuting and foraging (mean of 4.5 ± 1.5 sonar sound groups when the bats were foraging). All of the recordings at site B were approximately 4.2 s. The recordings at site A were shorter and of variable length as the bats flew straight out and did not circle around the roosting site.

### Flying bats produce sonar sound groups under different conditions in the lab

Here we compare the timing of calls produced by big brown bats across several conditions in the laboratory. Figure 4E shows the mean number of sound groups produced by the bat in the final 3 s of flight before a successful or failed attempt to capture the target (tethered mealworm or a flying praying mantis), in the open room, in the presence of clutter (plant) or with obstacles (nets) in the environment. Successful attempts are the trials in which the bat took the mealworm off the tether or captured the free-flying (deafened) mantis. Failed attempts are trials in which the bat produced the terminal buzz and hit the insect but either dropped it or failed to take it off the tether. The mean number of sonar sound groups per trial (3 s of data prior to the time of capture of the target) increased with an increase in complexity of the environment and the task. In the open room task, bats produced an average of 7.4 ± 2.1 sonar sound groups per trial. When clutter in the form of an artificial plant was introduced to the environment, the average number of sonar sound groups increased to 8.0 ± 2.4 sonar sound groups per trial. In the task where the bats tracked and captured a freely flying praying mantis, the mean number of sonar sound groups was, 9.3 ± 1.8 sonar sound groups per trial. And finally, in the dual task of obstacle avoidance (net hole) and prey capture, the mean number of sonar sound groups was 9.7 ± 4.7 sonar sound groups per trial. All numbers reported here are per trial.

#### Bat tracking an erratically moving target while resting on a platform

Field, net, plant and free-flight insect capture experiments all show that bats produce sonar call groups under conditions of clutter or dynamic target trajectory. Here we extend this work to explicitly test the hypothesis that bats actively control the timing of calls and produce an increased number of sonar call groups under conditions of target trajectory uncertainty.

#### Increase in sound group doublets and triplets with increase in uncertainty in target position

**Figure 5:**
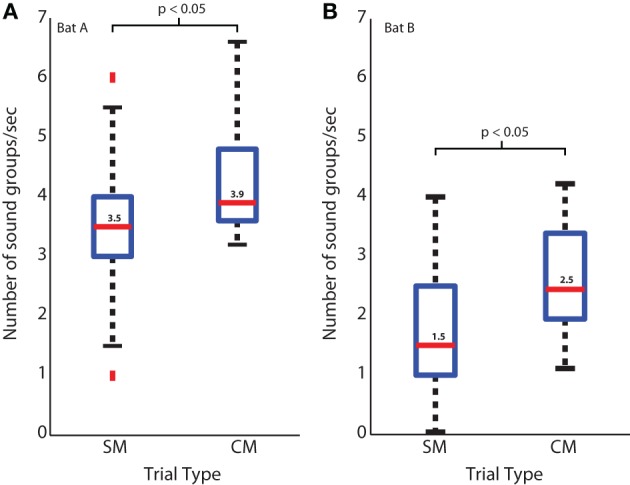
**Sound groups during simple and complex target motions. (A)** Bat A sound group usage for simple and complex target motion trials. Blue box represents the middle 50% of the data, red bar is the median. Black bars detail the range of the data, and red dots are outliers. **(B)** Same as in **(A)**, but for Bat B.

Box plots showing the number of sonar sound groups produced by bats tracking a target in the CM and SM trial sequences are displayed in Figures 5A,B for two bats, Bat A and Bat B, respectively. Both bats showed a significant decrease in the number of sonar sound groups in the sequence of SM trials, as the predictability of the target position increased in repeated SM trials, as compared to randomly introduced CM trials. The median number of sonar sound groups produced per unit time (seconds) for Bat A was 3.9 for the CM trials, which was significantly greater than the median of 3.5 for the SM trials (*p* < 0.05 Mann-Whitney *U*-test). The median of the number of sonar sound groups produced per unit time (seconds) for Bat B was 2.5 in the CM trials, which was significantly greater than the median of 1.5 in the SM trials (*p* < 0.05 Mann-Whitney *U*-test). Moreover, in instances when several SM trials were presented in sequence, the number of sonar sound groups produced by the bat decreased as trial-to-trial target trajectories became more predictable (data not shown). Box plots show the spread of the data.

#### Comparison of call group parameters across different conditions

**Figure 6:**
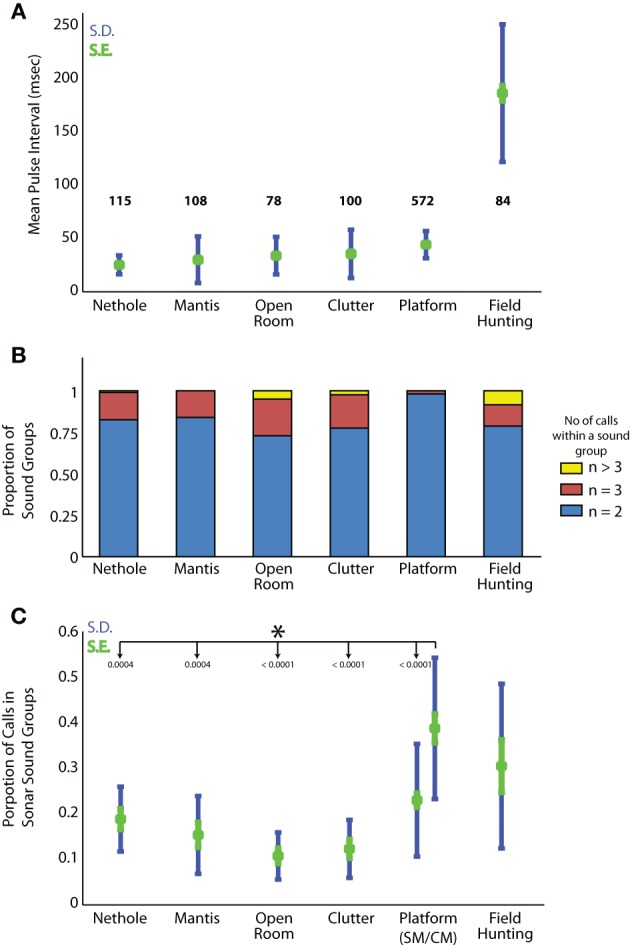
**Sound group parameters across conditions. (A)** Mean pulse interval time for sound groups in each experimental condition. Standard error in green, standard deviation in blue. The mean P.I. for bats hunting in the field is significantly greater than all other experiments. Refer to Table 3 for a pairwise comparison of the mean sonar sound group PI between conditions. **(B)** Proportion of 2, 3, or ≥3 call sound groups produced in each experimental condition. **(C)** Proportion of sounds produced by the bats in sonar sound groups as compared to the total number of sounds produced under different behavioral conditions. Standard error in green, standard deviation in blue.

In addition to producing sonar calls, as presented in Figures 4, 5, bats actively adjusted other sonar signal temporal parameters. Here we compare pulse intervals of sonar sound groups across different experimental conditions (Figure 6A). As noted above, commuting bats do not produce sound groups and therefore no data from recordings at field site A is included here. The average sound group PI (Pulse Interval) for bats flying under conditions of clutter was 35.4 ± s.e.m. of 7.2 ms. Average sound group PI for bats performing in the net hole and mantis experiments was 25.1 ± s.e.m. of 2.8 ms and 29.8 ± s.e.m. of 6.9 ms respectively. When the bat captured tethered mealworms in the open room condition, the average sound group PI was 33.6 ± s.e.m. of 6.2 ms. When the bat tracked tethered meal worms from a resting position on a platform, the average sound group PI was 44.7 ± s.e.m. of 0.5 ms. In field site B, the average sound group PI was 118.2 ± s.e.m. of 8.2 ms. Many of these pairwise comparisons of PI in different environments were significantly different from one another (Table 3). To summarize, PI's of experiments in the large flight room were comparable, but significantly less than the mean PI of sonar sound groups produced by bats in the platform experiment, while bats hunting in the field produced sonar sound groups with the largest PI's. The net hole experiment in the large flight room resulted in the shortest sonar sound group PI's, presumably because the room was partitioned into smaller quadrants for this experiment. Figure 6B compares the mean number of sounds in sound groups across the different conditions. Table 2 summarizes the proportion of time the bat produced sonar sound groups with 2, 3, or more than 3 sonar calls (*N* = 2, *N* = 3 or *N* > 3 respectively). Our data also indicates that on average bats produce sonar sound groups with more calls (*N* ≥ 3) in the field compared to the laboratory.

**Table 2:** **Number of sounds, 2, 3 or more than 3, calls contained in sound groups for each experimental condition**.

	**Clutter**	**Net hole**	**Mantis capture**	**Open space**	**Platform**	**Field-foraging**
*N* = 2	77.5	82.5	83.9	72.9	89.5	78.7
*N* = 3	20	16.4	16.1	22	10.5	12.8
*N* ≥ 4	2.5	1.1	0	5.1	0	8.5
Sample size (trials)	10	10	10	8	91	10

Values in percentages. For example, 89.5% of sound groups for the platform experiment were sound groups with 2 calls (doublets).

In one further set of analysis, we examined the bat's proportional use of sonar sound groups across laboratory tasks and field conditions. For this analysis, we compared the proportion of sonar pulses the bat's produced as part of sonar sound groups compared to the total number of sounds produced by the bat during each behavioral condition (Figure 6C). This analysis shows that the experimental condition with the highest proportional use of sonar sound groups was in the task in which the bat tracked a target moving back and forth (Complex Motion trials) from the platform. All foraging flight experiments in the laboratory and the field showed very similar sonar sound group production.

## Discussion

By comparing the echolocating bat's temporal control of sonar vocalizations in both field and laboratory settings, it is evident that bats increase the production of sonar sound groups when faced with challenging tasks, e.g., tracking and capturing a target with an unpredictable trajectory or taking prey in the presence of clutter. We found that when bats are foraging in the field, they produce sonar sound groups during the approach stages of insect capture, well before the terminal buzz, presumably because they require higher spatio-temporal localization accuracy to position an insect with a potentially erratic flight path. In contrast, when the bats are commuting from a roost to a foraging site, almost no sonar sound groups were recorded. These results parallel those found in the lab. When the bat is flying in an open flight room, comparatively few sonar sound groups are produced; but when the bat is catching tethered insects in the presence of acoustic clutter, there is an increase in the production of sonar sound groups. Furthermore, in the net hole experiment, where the bat had to shift its attention between an opening in the net and a more distant tethered insect, there was a large increase in the production of sonar sound groups. Lastly, we found that when the bat is tracking erratically moving prey items, either from a resting position on a platform or catching a flying insect on the wing, the prevalence of sonar sound groups increased significantly. Taken together, these results provide further evidence that bats actively produce sonar sound groups when faced with challenging spatial tasks.

It has been well documented that bats actively adjust a number of call parameters (sonar beam direction, frequency, intensity, duration, and interval) as they perform echolocation tasks in diverse settings (Schnitzler and Kalko, 2001; Ghose and Moss, 2003; Moss et al., 2006; Surlykke and Kalko, 2008; Chiu et al., 2009; Surlykke et al., 2009a,b; Aytekin et al., 2010; Brinkløv et al., 2010; Mantani et al., 2012; Jakobsen et al., 2013; Ratcliffe et al., 2013). The overarching goal of the current report is to re-examine the hypothesis that temporal patterning of sonar vocalizations is central to the bat's success at navigating and intercepting prey under complex laboratory and field conditions and to develop insight in to the perceptual consequences for the bat's production of sonar sound groups. In the sections below we attempt to shed light on some of the basic questions regarding sonar sound groups: (1) Do sonar sound groups have behavioral significance? (2) Under what circumstances do bats produce sound groups? (3) How does the bat adapt its sonar behavior to different environmental or clutter boundaries? (4) How might sonar sound groups perceptually sharpen spatio-temporal localization in bats? The answers to these questions will help us to advance our understanding of temporal processing in spatial perception by sonar in bats.

### Do bats actively produce sound groups to enhance information carried by echo returns?

One of the first and very important questions one must ask when examining the temporal patterning of sonar signals is whether call clustering has functional significance for the animal. In this context, we emphasize that the definition of sonar sound groups is arbitrary and defined by the researcher (see Moss et al., 2006), and should be updated as we learn more about sonar behavior to capture information that has behavioral relevance. Relevant to this point, we note that the average PI of sound groups in the field are much longer (115 ms) than in any condition in the lab (25–37 ms), which suggests that the environmental conditions directly influence the intervals of sonar sound groups used for spatial perception.

The data we have presented here provides evidence that bats actively produce sonar sound groups under task conditions that require spatio-temporal accuracy in tracking and figure ground segregation. Figure 4B shows that in the field when bats emerge from their roosts and are commuting to another site, they produce very few sonar sound groups. Feeding buzzes were never observed in this situation, indicating that the bats were not actively engaged in searching or tracking prey as they emerged from their roosts, and we infer that spatial localization requirements were low. In contrast, actively foraging bats produce a significantly greater number of sonar sound groups as they engage in goal-oriented tasks.

One way to test the functional importance of a behavior is to modify certain environmental parameters and then observe the animal's responses. The bat's echolocation behavior in the platform target tracking experiment reported here serves to illustrate how the bat actively produces sonar sound groups when it encounters uncertainty in the trajectory of the target (see Figure 5). The complex target trajectories (CM trials) were designed to have multiple back and forth motion (Figure 3A—red and black motion trajectories). A bat introduced to CM trials for the first time would experience uncertainty in the target's spatio-temporal position compared to the simple motion target trajectory on which the bat was initially trained. When the bat tracked the target moving with the CM trajectory it increased the number of sound groups produced per unit time (seconds) (Figure 5) as compared to when the bat tracked the target with repeated SM trajectories. This experiment therefore provides direct evidence that changing the complexity and uncertainty of the moving target changes the bat's echolocation behavior, indicating that temporal patterning of sonar vocalization is a strategy employed by the big brown bat to improve its spatio-temporal resolution of an uncertain target's position.

### De-coupling sonar sound groups from wing beat strokes

The production of sonar calls can be energetically expensive and hence coupling sonar calls with the upstroke of the wing beat cycle, and therefore coinciding with exhalation (Suthers et al., 1972) can help reduce the energy cost of sonar vocalizations (Speakman et al., 1989; Speakman and Racey, 1991). A previous study by Moss et al. (2006) examined the relation between sonar call production and wing beat. The results indicate that for sonar vocalizations of freely flying bats in the laboratory, calls with pulse intervals larger than 70 ms were coupled to the upstroke of the wingbeat, but for PIs shorter than 70 ms, call timing occurred across different phases of the wingbeat cycle (see Moss et al., 2006, for more details). In this earlier study, however, analysis included only measurements of the peak and trough of the bat's wing beat cycle. Because the bat's wing beat can show asymmetries in the up/down stroke excursion, it is important to look more closely at the relation between sonar sound group production and wing beat.

Koblitz et al. (2010) examined emission times of sonar sound groups and their coupling with different phases of wing beat in the big brown bat. Their results indicate that the emission of sonar sound groups has a tri-modal distribution. The first call of the sound group occurs at the end of the down stroke, the center of the sound group occurs when the wings are horizontal and the last call of the sound group occurs at the end of the upstroke. In this study the bats were trained to fly across a room without any obstacles or acoustic clutter. In future research, it would be interesting to analyze the relation between the sonar sound group emission patterns and wing beat when a bat is performing complex flight maneuvers.

In the experiment reported here in which bats tracked a moving target from a stationary position on a platform, sonar sound groups were prominent (Figure 5). Obviously, wing beat is completely absent in bats echolocating from a platform; however, bats would be expected to coordinate their sonar call production with respiration to optimize on energy consumption. We have not measured the respiration of bats while they perform the tracking task on the platform, and this could be investigated in future experiments.

### Spatially-guided behavior

The data presented in this report suggest that echolocating bats increase sonar sound group production in the context of spatially challenging behaviors. When a bat is flying in an open room in the laboratory, sonar sound group production is relatively low. When the bat is navigating through obstacles, however, sonar sound groups are produced as the bat inspects each opening in a net through which it can fly to gain access to a food reward. This comparison suggests that sound group production is not used solely in the context of hunting, but is also employed when the bat is negotiating obstacles. These laboratory results are consistent with data from field recordings. Furthermore, bats hunting in the field sometimes, but not always, produce sound groups just prior to the buzz phase, indicating that this call pattern may be important for target capture. By contrast, bats commuting in a familiar environment produce very few sound groups. This comparison offers another demonstration of how a bat increases sound group production during goal-directed behaviors, but not during routine commuting flight. Furthermore, considering that sonar sound group production increases under challenging conditions (i.e., spatial navigation around obstacles, insect capture), we provide evidence that sonar sound groups are used actively by bats when they attempt to gather more detailed information about the location of objects in the environment. This idea is supported by the finding that bats used sonar sound groups most frequently when it tracked the complex motion of the target from a resting position on a platform. The complex motion tracking condition may capture some of the target uncertainty a bat encounters in the field as it pursues insects engaged in evasive maneuvers.

### Temporal control over sonar calls varies with task and environmental complexity

In this article we have presented evidence of temporal clustering of sonar calls when bats are engaged in a variety of tasks, both in the lab and the field, when they are flying freely or tracking an unpredictably moving target from a stationary position on the platform. An important question that arises is whether bats vary the properties of sonar sound groups across different environmental conditions and task complexities. In this section we compare and further analyze the data presented in Figure 6 to show that bats indeed modify sonar sound group parameters with environment and task conditions. Most noteworthy are the differences in the prevalence of sound group production, the number of sounds in a group, and the pulse interval of calls in a group, all of which appear to be related to the uncertainty of the target trajectory, figure-ground segregation, and the environment in which the bat echolocates.

#### Prevalence of sonar sound groups changes according to uncertainty of target trajectory

Sonar call groups were produced by bats as they foraged in the field and the laboratory. Our interpretation of this result is that the bat increases sound group production to more accurately resolve the location of the insect from the clutter. This interpretation is further corroborated by the laboratory studies that placed different demands on the bat's spatial localization by sonar. Specifically, when a bat tracked a moving prey item from a resting position on a platform, its sonar sound group production increased when the target trajectory was unpredictable. When the insect moved toward the bat with a simple and already familiar velocity path, the bat produced very few sonar sound groups. In contrast, when the bat tracked an insect that moved back and forth with changing velocities and directions, sonar sound group production increased significantly (see Figure 5A). This result suggests that the echolocating bat actively controls the timing of its calls to track an erratically moving target.

#### Sonar sound groups help bats separate figure and ground

*Eptesicus fuscus* has been observed hunting near vegetation (Simmons et al., 2001). To be successful foragers, bats hunting in cluttered environments must be able to discriminate between acoustic clutter resulting from vegetation and their desired targets. Our results (Figure 4) indicate that in the experiments when bats had to capture tethered mealworms placed near an artificial plant or in the experiment in which bats were required to localize an insect behind an opening in a mist net, the animals increased the rate of sonar sound group production.

#### Bats scale the PI of sonar sound groups according to the boundaries of their immediate environment

Modulating PI can be an effective strategy to avoid mixing of calls and echoes from distant clutter, which may represent the effective boundary of the bat's active space. A survey of field site B indicates that a bat following a stereotypical flight trajectory would on average be at a distance from the boundaries (thicket of trees) that is approximately four times the distance from boundaries (walls, ceiling and floor) in the laboratory. The average PI (Figure 6A) of all the sonar sound group recordings from field site B is about 185 ± 27 ms. This scales well with the boundaries of the foraging site. In the laboratory study of the bat resting on the platform and tracking an erratically moving target, the distance of the bat from the far wall was 5 m. To allow sufficient time for an entire echo stream to arrive from objects distributed along a range axis of 5 m, a bat would wait 30 ms before producing its next call in the sound group, and the average PI would be maintained above 30 ms (Figure 6A). A comparison of the sound group PI's when the bat is stationary on the platform and tracking a moving target to the sound group PI's produced by the bat when it is flying under different conditions in the laboratory offers strong evidence that bats adjust the PI of their sound groups to the boundaries of their immediate environment (Figure 6A and Table 3). A closer examination of the average distance of the bat from the boundaries in each of these experiments (platform compared to the laboratory flight experiments) reveals that in the prey tracking experiment, the bat on the platform is approximately 5 m from the wall, while in the laboratory flight experiments, the bat typically flies through the middle of the room with an average distance of less than 3 meters from the nearest wall (see Figure 2 for schematics of each experimental flight room). From the laboratory to the field, the boundaries of the environment increased by a factor of 4, which is approximately the same factor by which the PI is scaled. Our data suggests that the bat tends to cluster its calls when it is actively tracking an object of interest, and the PI of the sound group is adjusted by the bat according to the environment in which it operates.

**Table 3:** ***p*-values for pairwise, two-tailed *T*-tests performed on the sonar sound group PI data reported in Figure 6A**.

	**Field hunting**	**Platform**	**Open room**	**Mantis**	**Net hole**
Clutter	<10^−10^	<0.00005	0.58	0.07	<0.001
Net hole	<10^−10^	<0.00005	<0.001	0.33	
Mantis	<10^−10^	<0.00005	0.2		
Open room	<10^−10^	<0.00005			
Platform	<10^−10^				
Field hunting					

A Bonferroni correction was performed to account for multiple comparisons, resulting in a p-value threshold of 0.0033.

A recent study by Hiryu et al. (2010) showed that under extreme clutter conditions in which the bat reduced its PI to below that set by the environmental boundary (also referred to as the “outer window,” see Wilson and Moss, 2004), it employs a different strategy to disambiguate echo streams between two calls within a sonar sound group. In their study, the bat shifted frequencies of calls within a sonar call doublet to enable assignment between calls and cascades of echoes in a highly cluttered environment. In most settings, bats adjust the pulse interval of sonar sound groups to avoid overlap of echo streams. However, under extreme clutter conditions, bats shift frequencies of calls within sound groups to disambiguate echo streams (Hiryu et al., 2010). Here we see that when bats do not adjust call group PI to the environmental boundaries, they adopt additional vocal strategies to support spatial perception by sonar.

#### The number of calls per sonar sound group depends on the task and environment

Another observation that may contribute to our understanding of the functional importance of sonar call timing to spatial resolution of the environment is the number of sounds contained in groups (doublets, triplets or higher order sonar sound groups) we observed under different conditions. The two extremes are the platform, where we rarely observe sonar sound groups with three or more calls, and the field where we frequently observe sonar sound groups with more than four or five (see Figure 6B). When bats flew in the laboratory flight room, we typically observed sound groups with two, three or four. As we have a comparatively few trials for the flight conditions, we do not have enough statistical power to test significance (Figure 6B). However, we hypothesize that the bat adapts the number of sonar sounds per sonar sound groups according to its immediate environment and its challenges. Future experiments with a greater number of recordings should be able to elucidate this further.

Data from many different studies demonstrate that sonar sound group production occurs at times when spatio-temporal localization demands are high. Bats increase the prevalence of sonar sound groups when they are tracking erratically moving prey, when trying to resolve target from clutter, and when navigating complex scenes. For each of these behavioral situations, the bats produced sonar sound groups at times when increased spatial resolution was paramount for success.

Here we consider why sonar sound group production may help the bat to localize and track an object. When a bat is tracking a moving insect, computing the distance and velocity of the insect involves computations of the insect's position with respect to the bat over longer temporal windows. The production of sonar call doublets may serve two purposes: (1), increase the echo return rate over a restricted time window, which may serve to increase the reliability of echo reception by the sonar receiver. (2) By keeping the pulse interval stable, as in the case of sonar sound groups, the bat receives echo updates with a regular periodicity, which may allow the bat to more easily assign different echo streams to the original sonar pulses. The same idea applies to a bat navigating a complex maze or when the environment is full of acoustic clutter and many objects are reflecting echoes. In all of these contexts, sampling information from the environment is simplified by stable temporal timing of sensory updates.

In conclusion, this study of the echolocating big brown bat in a number of different tasks and acoustic situations of varying complexity, demonstrates that that this animal employs temporal control of its sonar calls to effectively probe its sensory world. In more simple acoustic environments, the bat tends to monotonically decrease pulse interval with respect to target distance. Conversely, when the bat is placed in a more dynamic and complex environment, it temporally organizes its sonar vocalizations into sound groups, which are structured to provide periodic updates about the sensory world. The increase in sonar sound group production is not limited to instances of hunting, since bats navigating obstacles also produce sound groups, which may aid in building a detailed representation of the environment. The results of this study motivate further experiments and models examining how the timing of sensory signals may shape perception.

### Conflict of interest statement

The authors declare that the research was conducted in the absence of any commercial or financial relationships that could be construed as a potential conflict of interest.

## References

[B1a] Agosta S. J. (2002). Habitat use, diet and roost selection by the Big Brown Bat (Eptesicus fuscus) in North America: a case for conserving an abundant species. Mamm. Rev. 32, 179–198 10.1046/j.1365-2907.2002.00103.x

[B1] Aytekin M. Mao B. Moss C. F. (2010). Spatial perception and adaptive sonar behavior. J. Acoust. Soc. Am. 128, 3788–3798 10.1121/1.3504707 21218910 PMC3037775

[B2] Brinkløv S. Kalko E. K. V. Surlykke A. (2010). Dynamic adjustment of biosonar intensity to habitat clutter in the bat *Macrophyllum macrophyllum* (*Phyllostomidae*). Behav. Ecol. Sociobiol. 64, 1867–1874 10.1007/s00265-010-0998-9

[B2a] Brigham R. M. Fenton M. B. (1986). The influence of roost closure on the roosting and foraging behaviour of Eptesicus fuscus (Chiroptera: Vespertilionidae). Can. J. Zool. 64, 1128–1133 10.1139/z86-169

[B3] Chiu C. Xian W. Moss C. F. (2009). Adaptive echolocation behavior in bats for the analysis of auditory scenes. J. Exp. Biol. 212, 1392–1404 10.1242/jeb.027045 19376960 PMC2726850

[B4] Ghose K. Moss C. F. (2003). The sonar beam pattern of a flying bat as it tracks tethered insects. J. Acoust. Soc. Am. 114, 1120 10.1121/1.1589754 12942989 PMC3384009

[B5] Ghose K. Moss C. F. (2006). Steering by hearing: a bat's acoustic gaze is linked to its flight motor output by a delayed, adaptive linear law. J. Neurosci. 26, 1704–1710 10.1523/JNEUROSCI.4315-05.2006 16467518 PMC3437256

[B6] Ghose K. Triblehorn J. D. Bohn K. Yager D. D. Moss C. F. (2009). Behavioral responses of big brown bats to dives by praying mantises. J. Exp. Biol. 212, 693–703 10.1242/jeb.019380 19218521 PMC2726853

[B7] Griffin D. R. (1958). Listening in the Dark: The Acoustic Orientation of Bats and Men. New Haven, CT: Yale University Press Available online at: http://psycnet.apa.org/psycinfo/1959-07554-000

[B8] Griffin D. R. Webster F. A. Michael C. R. (1960). The echolocation of flying insects by bats. Anim. Behav. 8, 141–154 10.1016/0003-3472(60)90022-1

[B9] Hayward B. Davis R. (1964). Flight speeds in western bats. J. Mammal. 45, 236–242 10.2307/137698618457938

[B10] Hiryu S. Bates M. E. Simmons J. A. Riquimaroux H. (2010). FM echolocating bats shift frequencies to avoid broadcast-echo ambiguity in clutter. Proc. Natl. Acad. Sci. U.S.A. 107, 7048–7053 10.1073/pnas.1000429107 20351291 PMC2872447

[B11] Jakobsen L. Brinkløv S. Surlykke A. (2013). Intensity and directionality of bat echolocation signals. Front. Physiol. 4:89 10.3389/fphys.2013.00089 23630501 PMC3635024

[B12] Jen P. H.-S. McCarty J. K. (1978). Bats avoid moving objects more successfully than stationary ones. Nature 275, 743–744 10.1038/275743a0 703838

[B12a] Jones G. Holderied M. W. (2007). Bat echolocation calls: adaptation and convergent evolution. Proc. Biol. Sci. 274, 905–912 10.1098/rspb.2006.0200 17251105 PMC1919403

[B13] Kalko E. K. V. (1995). Insect pursuit, prey capture and echolocation in pipestirelle bats (*Microchiroptera*). Anim. Behav. 50, 861–880 10.1016/0003-3472(95)80090-5

[B14] Kalko E. K. V. Schnitzler H.-U. (1989). The echolocation and hunting behavior of Daubenton's bat, Myotis daubentoni. Behav. Ecol. Sociobiol. 24, 225–238 10.1007/BF00295202

[B15] Kalko E. V. Schnitzler H.-U. (1993). Plasticity in echolocation signals of European pipistrelle bats in search flight: implications for habitat use and prey detection. Behav. Ecol. Sociobiol. 33, 415–428 10.1007/BF00170257

[B16] Koblitz J. C. Stilz P. Schnitzler H.-U. (2010). Source levels of echolocation signals vary in correlation with wingbeat cycle in landing big brown bats (*Eptesicus fuscus*). J. Exp. Biol. 213, 3263–3268 10.1242/jeb.045450 20833918

[B17] Mantani S. Hiryu S. Fujioka E. Matsuta N. Riquimaroux H. Watanabe Y. (2012). Echolocation behavior of the Japanese horseshoe bat in pursuit of fluttering prey. J. Comp. Physiol. A Neuroethol. Sens. Neural. Behav. Physiol. 198, 741–751 10.1007/s00359-012-0744-z 22777677

[B18] Moss C. F. Bohn K. Gilkenson H. Surlykke A. (2006). Active listening for spatial orientation in a complex auditory scene. PLoS Biol. 4:e79 10.1371/journal.pbio.0040079 16509770 PMC1393756

[B19] Moss C. F. Schnitzler H.-U. (1995). Behavioral studies of auditory information processing, in Hearing by Bats, eds Popper A. N. Fay R. R. (New York, NY: Springer), 87–145

[B20] Moss C. F. Surlykke A. (2001). Auditory scene analysis by echolocation in bats. J. Acoust. Soc. Am. 110, 2207 10.1121/1.1398051 11681397

[B21] Moss C. F. Surlykke A. (2010). Probing the natural scene by echolocation in bats. Front. Behav. Neurosci. 4:33 10.3389/fnbeh.2010.00033 20740076 PMC2927269

[B22] Petrites A. E. Eng O. S. Mowlds D. S. Simmons J. A. DeLong C. M. (2009). Interpulse interval modulation by echolocating big brown bats (*Eptesicus fuscus*) in different densities of obstacle clutter. J. Comp. Physiol. A Neuroethol. Sens. Neural. Behav. Physiol. 195, 603–617 10.1007/s00359-009-0435-6 19322570

[B23] Ratcliffe J. M. Elemans C. P. H. Jakobsen L. Surlykke A. (2013). How the bat got its buzz. Biol. Lett. 9:20121031 10.1098/rsbl.2012.1031 23302868 PMC3639754

[B24] Schnitzler H.-U. Kalko E. K. V. (2001). Echolocation by insect-eating bats. Bioscience 51:557 10.1641/0006-3568(2001)051[0557:EBIEB]2.0.CO;2

[B24a] Siemers B. M. Schnitzler H.-U. (2004). Echolocation signals reflect niche differentiation in five sympatric congeneric bat species. Nature 429, 657–661 10.1038/nature02547 15190352

[B25] Simmons J. A. Eastman K. M. Horowitz S. S. O'Farrell M. J. Lee D. N. (2001). Versatility of biosonar in the big brown bat, *Eptesicus fuscus*. Acoust. Res. Lett. Online 2, 43 10.1121/1.1352717

[B26] Simmons J. A. Fenton M. B. O'Farrell M. J. (1979). Echolocation and pursuit of prey by bats. Science 203, 16–21 10.1126/science.758674 758674

[B27] Speakman J. R. Anderson M. E. Racey P. A. (1989). The energy cost of echolocation in pipistrelle bats (*Pipistrettus pipistrellus*). J. Comp. Physiol. A 165, 679–685 10.1007/BF00610999

[B28] Speakman J. R. Racey P. A. (1991). No cost of echolocation for bats in flight. Nature 350, 421–423 10.1038/350421a0 2011191

[B29] Surlykke A. Boel Pedersen S. Jakobsen L. (2009a). Echolocating bats emit a highly directional sonar sound beam in the field. Proc. Biol. Sci. 276, 853–860 10.1098/rspb.2008.1505 19129126 PMC2664374

[B30] Surlykke A. Ghose K. Moss C. F. (2009b). Acoustic scanning of natural scenes by echolocation in the big brown bat, *Eptesicus fuscus*. J. Exp. Biol. 212, 1011–1020 10.1242/jeb.024620 19282498 PMC2726860

[B31] Surlykke A. Kalko E. K. V (2008). Echolocating bats cry out loud to detect their prey. PLoS ONE 3:e2036 10.1371/journal.pone.0002036 18446226 PMC2323577

[B32] Surlykke A. Moss C. F. (2000). Echolocation behavior of big brown bats, *Eptesicus fuscus*, in the field and the laboratory. J. Acoust. Soc. Am. 108, 2419–2429 10.1121/1.1315295 11108382

[B33] Suthers R. A. Thomas S. P. Suthers B. J. (1972). Respiration, wing-beat and ultrasonic pulse emission in an echo-locating bat. J. Exp. Biol. 56, 37–48

[B34] Triblehorn J. D. Ghose K. Bohn K. Moss C. F. Yager D. D. (2008). Free-flight encounters between praying mantids (*Parasphendale agrionina*) and bats (*Eptesicus fuscus*). J. Exp. Biol. 211, 555–562 10.1242/jeb.005736 18245632

[B35] Wilson W.W. Moss C. F. (2004). Sensory-motor behavior of free-flying FM bats during target capture, in Advances in the Study of Echolocation in Bats and Dolphins, ed Thomas J. A. (Chicago, IL: University of Chicago Press), 22–27

[B36] Wong J. G. Waters D. A. (2001). The synchronisation of signal emission with wingbeat during the approach phase in soprano pipistrelles (*Pipistrellus pygmaeus*). J. Exp. Biol. 204, 575–583 Available online at: http://www.ncbi.nlm.nih.gov/pubmed/11171308 11171308 10.1242/jeb.204.3.575

